# Role of Spinal Surgery Drainage Techniques in Postoperative Outcomes: Insights From a Comprehensive Literature Review

**DOI:** 10.7759/cureus.69636

**Published:** 2024-09-18

**Authors:** Wamedh E Matti, Hussain J Kadhim, Ahmed M Taha, Maher K Mustafa, Rasha A Alshakarchy, Rania H Al-Taie, Mustafa Ismail

**Affiliations:** 1 Department of Neurosurgery, Neuroscience Hospital, Baghdad, IRQ; 2 Department of Neurosurgery, Neurosurgery Hospital, Baghdad, IRQ; 3 Department of Neurosurgery, Fallujah Teaching Hospital, Anbar, IRQ; 4 Department of Surgery, College of Medicine, University of Mustansiriyah, Baghdad, IRQ; 5 Department of Surgery, Baghdad Teaching Hospital, Medical City Complex, Baghdad, IRQ

**Keywords:** hematoma formation, infection rates, postoperative outcomes, spinal drains, spinal surgery

## Abstract

Postoperative management often demands the introduction of several strategies in an attempt to minimize complication rates. One of the routine strategies includes the use of spinal drains, which have been questioned for their efficacy in improving postoperative outcomes. However, its role in postoperative outcomes is still debated. In general, this elucidation of an extensive literature review supports the synthesis of current evidence regarding the role of spinal drains in infection rates, hematoma formation, and overall patient recovery. A comprehensive search of PubMed from 2000 to 2024 was performed, focusing on studies investigating the use of spinal drains in spinal surgeries and their associated postoperative outcomes. It followed the guidelines outlined by the Preferred Reporting Items for Systematic Reviews and Meta-Analyses (PRISMA). The inclusion criteria were studies related to spinal surgeries, excluding case reports, reviews, and editorials, and limited to articles published in English. Quality assessment was performed using the Risk Of Bias In Non-randomized Studies of Interventions (ROBINS-I) tool.

A total of 19 studies were included, with different designs and varied sample sizes. The sample size was from 25 to 2,446 patients. Findings on infection rates were mixed; while one group of studies showed no significant differences in patients with and without drains, another group showed a reduced rate of reoperation for surgical site infections in patients with drains. In general, hematoma formation rates were reported to be the same across groups, while a few studies indicated that drains were more effective in managing wound exudates compared to no drains. Recovery outcomes indicated that patients who had a wound drain were more likely to stay in the hospital longer, although an improvement was noticed with time-driven wound drain removal, which resulted in shorter hospital stays and earlier ambulation. The use of spinal drains in postoperative spinal surgery presents both benefits and drawbacks. Spinal drains can assist in the management of wound exudates, and earlier detection of infection complications increases hospital stays and complications. Indeed, whether to use spinal drains or not should be an individual decision, weighing the potential benefits and risks. Future studies need to be done in order to establish clear guidelines for the use of drainage systems in various spinal surgical cases.

## Introduction and background

In spinal surgeries, postoperative management is often directed toward the prevention of post-surgical complications such as epidural hematomas and surgical site infections. In this regard, the use of spinal drains with closed suction drainage systems has been widely adopted to reduce such adverse outcomes. Nevertheless, the role that spinal drains play in influencing postoperative outcomes remains controversial and debated by surgeons and researchers alike [1]. Over the years, there has been continuous debate about surgical drains being effective in spine surgeries and, at the same time, bringing down postoperative complications like hematoma formation or infection. Some studies have found that despite the quite common use of drains with the intent to minimize hematoma risks, the care in preventing postoperative complications remains questionable. For instance, there is evidence that the use of drains does not notably decrease the rates of postoperative hematomas or infections after spine surgery [1]. However, occlusive dressings have been supported based on the benefit of maintaining the wound free of infection and promoting better wound healing, although this evidence is variable [2].

Although it is rare, epidural hematoma can cause serious neurological sequelae, such as motor weakness, bowel and bladder dysfunction, and even paralysis. There have been a few studies that show the incidence rate of symptomatic epidural hematoma after spinal surgeries lies between 0.1% and 0.24% [3, 4]. The closed suction drainage system in spinal surgeries is aimed at reducing these risks by decreasing the amount of hematoma formation since it can act as a culture medium that allows the growth and proliferation of bacteria, which would lead to infections. Thus, this research aims to evaluate whether surgical drains are of any significant necessity or efficacy in spinal surgeries. While some studies would have them included because of the associated benefits in reducing hematoma and the ability to detect early infections, others emphasized the risks associated with an increased rate of infections [3]. Therefore, the literature review was conducted to outline current evidence on the role of spinal drains in postoperative outcomes regarding infection rates, hematoma formation, and general recovery of patients. 

## Review

Methods

Study Design

**Figure 1:**
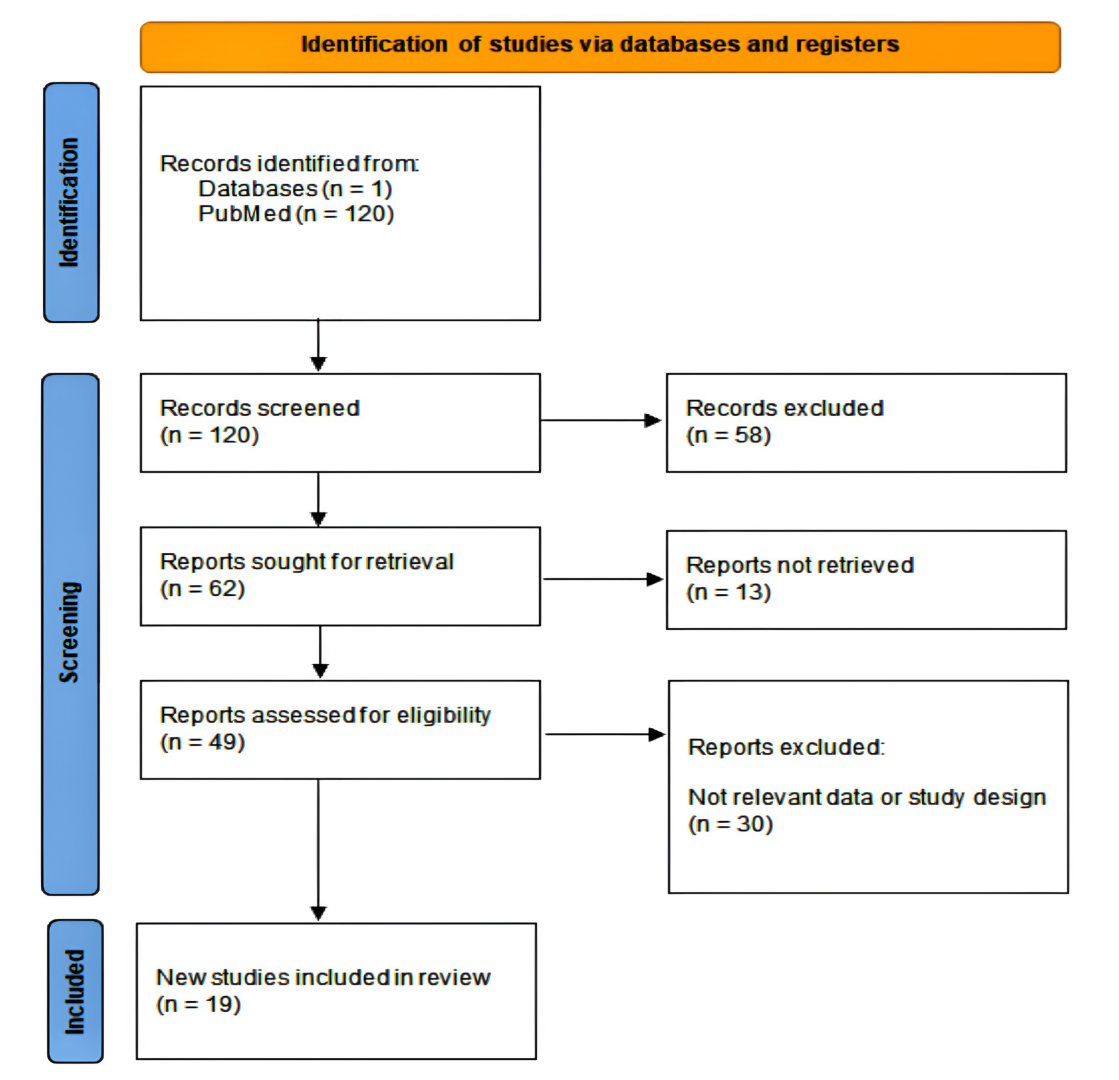
A PRISMA flow diagram showing the study selection process for studies on drains and dressings in spine surgery PRISMA: Preferred Reporting Items for Systematic Reviews and Meta-Analyses

This comprehensive literature review was conducted to assess the role of spinal drains in postoperative outcomes, specifically focusing on infection rates, hematoma formation, and overall patient recovery. It followed the guidelines outlined by the Preferred Reporting Items for Systematic Reviews and Meta-Analyses (PRISMA) (Figure 1) [5]. The review included studies published in peer-reviewed journals that investigated the use of spinal drains in various spinal surgeries. A systematic search of the literature was performed using the PubMed database. The search terms included "drain" AND" "spinal surgery." The search was limited to articles published in English from 2000 to 2024.

Inclusion and Exclusion Criteria

Studies were included if they investigated the use of spinal drains in spinal surgeries, reported on postoperative outcomes such as infection rates, hematoma formation, and patient recovery, and included a control group without the use of spinal drains for comparison.

Studies were excluded if they did not provide the mentioned data. Case reports, reviews, editorials, and non-English publications were excluded.

Data Extraction

Data were extracted independently by two reviewers (W.M. and R.A.) using a standardized form. The extracted data included study characteristics (authors, year of publication, country, study design), patient demographic information, type of drain used, duration of drain use, and postoperative outcomes (infection rates, hematoma formation, length of hospital stay, patient recovery metrics). Discrepancies between reviewers were resolved through discussion, and a third reviewer (M.I.) was consulted if necessary.

Quality Assessment

The quality of the included studies was assessed using the Risk Of Bias In Non-randomized Studies of Interventions (ROBINS-I) tool [6]. The ROBINS-I assessment was conducted independently by two reviewers. Each study was rated across domains, resulting in an overall risk of bias judgment (Table 1) [3,4,7-23].

**Table 1:** ROBINS-I assessment of the included studies ROBINS-I: Risk Of Bias In Non-randomized Studies of Interventions

Authors	Confounding	Selection of patients	Classification of interventions	Deviations from intended interventions	Missing data	Measurement of outcomes	Selection of reported results
Choi et al. (2016) [3]	Moderate	Low	Low	Low	Low	Low	Moderate
Kobayashi et al. (2015) [4]	Moderate	Low	Low	Low	Low	Low	Moderate
Poorman et al. (2014) [7]	Moderate	Low	Low	Low	Low	Low	Moderate
Herrick et al. (2018) [8]	Moderate	Low	Low	Low	Low	Low	Moderate
Elfiky et al. (2023) [9]	Moderate	Low	Low	Low	Low	Low	Moderate
Cabrera et al. (2023) [10]	Moderate	Low	Low	Low	Low	Low	Moderate
Liu et al. (2016) [11]	Moderate	Low	Low	Low	Moderate	Low	Moderate
Walid et al. (2012) [12]	Moderate	Low	Low	Moderate	Low	Low	Moderate
Kim et al. (2023) [13]	Moderate	Low	Low	Low	Low	Low	Moderate
Chen et al. (2018) [14]	Moderate	Low	Low	Low	Low	Low	Moderate
Shi et al. (2021) [15]	Moderate	Low	Low	Low	Low	Low	Moderate
von Eckardstein et al. (2015) [16]	Moderate	Low	Low	Low	Low	Low	Moderate
Pennington et al. (2019) [17]	Moderate	Low	Low	Moderate	Low	Low	Moderate
Gubin et al. (2018) [18]	Moderate	Low	Low	Low	Low	Low	Moderate
Adogwa et al. (2018) [19]	Moderate	Low	Low	Moderate	Low	Low	Moderate
Pivazyan et al. (2023) [20]	Moderate	Low	Low	Moderate	Low	Low	Moderate
Liang et al. (2020) [21]	Moderate	Low	Low	Low	Low	Low	Moderate
Armaghani et al. (2019) [22]	Moderate	Low	Low	Low	Moderate	Low	Moderate
Brown (2004) [23]	Moderate	Low	Low	Low	Low	Low	Moderate

Ethical Considerations

As this study is a literature review, it did not involve direct interaction with human participants or the collection of primary data. Therefore, ethical approval was not required.

Limitations

Potential limitations of this review include the heterogeneity of included studies, variations in surgical techniques, and differences in postoperative care protocols. These factors could affect the generalizability of the findings.

Results

Study Characteristics

A total of 19 studies were included in this comprehensive literature review, encompassing a variety of study designs such as retrospective case-control studies, prospective randomized studies, systematic reviews, and meta-analyses (Table 2) [3,4,7-23]. The sample sizes ranged from 25 to 2,446 patients, with studies conducted across various countries, including the USA, China, South Korea, Egypt, and others.

**Table 2:** Characteristics of the included studies on the use of drains and dressings in spine surgery SSI: surgical site infection; PPV: positive predictive value; NPV: negative predictive value; TXA: tranexamic acid; MIC: minimum inhibitory concentration

Author(s)	Country	Year	Study design	Sample size	Patient demographics	Type of surgery	Key findings
Choi et al. [3]	South Korea	2016	Retrospective study	70	34 men, 36 women, mean age 48.19 years	Single-level lumbar discectomy	Surgical drains did not elevate postoperative infection; Drain tip cultures allowed early detection of infection leading to faster antibiotic treatment
Kobayashi et al. [4]	Japan	2017	Retrospective cohort study	329	Adults, 54.4% female	Various spinal surgeries (cervical, thoracic, lumbar, sacral)	Drain tip cultures useful for early detection of SSI, especially methicillin-resistant bacteria; overall low PPV and high NPV for predicting wound infection
Poorman et al. [7]	USA	2014	Retrospective case-control	81	Adult patients	One- and two-level cervical spine fusions	No significant difference in complications, but longer operative time and hospital stay in drain group
Herrick et al. [8]	USA	2018	Multicenter retrospective study	1799	Adult patients	Posterior cervical decompression with instrumentation	Drains not associated with lower reoperation for hematoma, but may reduce SSI reoperations
Elfiky et al. [9]	Egypt	2022	Prospective randomized study	62	Age range 23-69, 51.6% female	Single-level posterior lumbar interbody fusion (PLIF)	Natural drainage reduced total blood loss compared to negative drainage without significant differences in postoperative outcomes
Cabrera et al. [10]	Various	2023	Cross-sectional survey	231	Surgeons 95.2% male, ages 25-65+	Open lumbar fusion surgery for degenerative pathologies	Most spine surgeons worldwide prefer to place a subfascial wound drain for degenerative open lumbar surgery, with removal based on time (mostly two days) or output criteria
Liu et al. [11]	China	2016	Meta-analysis	1904	Not specified	Posterior spinal surgery (various procedures)	No obvious evidence to support the application of closed suction drains for posterior spinal surgery. Drainage did not reduce infection, hematoma, or postoperative neurological injury.
Walid et al. [12]	USA	2012	Retrospective study	402	Mean age 57.3 years, 57% female, BMI 31.3 kg/m², 29.1% diabetic	Lumbar decompression and fusion (LDF)	Drain use did not significantly increase the risk of wound infection; Increased prevalence of postoperative fever and need for blood transfusion in drained group; No significant economic impact on hospital length of stay or charges except in lateral procedures
Kim et al. [13]	South Korea	2023	Retrospective study	1415	Mean age 64.9 years, 49% male	Cervical, lumbosacral, and thoracic spine surgeries	Drain tip cultures not useful for predicting SSI due to low positive predictive value; High positivity rate in SSI group
Chen et al. [14]	China	2018	Retrospective study	1125	17 women, 9 men, mean age 62.12 ± 10.42 years, mean BMI 28.88 ± 2.90 kg/m²	Lumbar spine surgery (discectomy, decompression, instrumented fusion)	No significant differences between single-tube and double-tube drainage methods in most aspects; Single-tube drainage group had better clinical outcomes and shorter hospital stays
Shi et al. [15]	China	2021	Case-control study	743	Comparable demographic characteristics (age, gender, BMI, medical history)	Posterior one-level or two-level lumbar fusion with instrumentation	Time-driven wound drain removal is associated with less postoperative drain output, less total blood loss, earlier ambulation, and shorter hospital stay compared to output-driven removal, without increasing the incidence of SSI or symptomatic spinal epidural hematoma (SHE).
Eckardstein et al. [16]	Germany	2015	Survey study	163	Not applicable (survey of surgeons)	Various spinal surgeries	Factors influencing drain use include type of surgery, size of wound, hemostasis at the end of procedure, and use of anticoagulatory drugs. Use of drains in spine surgery is with no clear guidelines. Most drains are discontinued by day 4, with time-driven removal more common in less invasive surgeries and output-driven removal in more invasive procedures.
Pennington et al. [17]	UK	2019	Retrospective cohort study	38	Adult patients, greater BMI, more likely to have diabetes and hypertension	Posterior spinal fusion	No clear benefit of closed suction drains in reducing infection or hematoma rates; higher transfusion rates in drain group
Gubin et al. [18]	Russia	2018	Randomized open-label trial	155	Adults (18-80 years)	Multi-level posterior spinal surgery	No-drain group had lower perioperative blood loss and transfusion requirements but higher postoperative aspirations
Adogwa et al. [19]	USA	2018	Retrospective cohort study	139	Adult spinal deformity patients	Spinal decompression and fusion	Use of postoperative subfascial drains may not reduce SSI or hematoma formation rates; associated with higher intraoperative blood loss and longer hospital stays
Pivazyan et al. [20]	Armenia	2023	Systematic review and meta-analysis	2446	Adult patients	Posterior spinal surgery	Prolonged prophylactic systemic antibiotics do not significantly reduce SSI rates in patients with closed suction drains after posterior spinal surgery
Liang et al. [21]	China	2019	Retrospective clinical trial	60	Adult patients with degenerative lumbar scoliosis	Posterior lumbar decompression and fusion of 3+ levels	Topical injection of TXA via drain and drain-clamping reduces postoperative blood loss and hospital stay in degenerative lumbar scoliosis surgery without increasing complications
Armaghani et al. [22]	USA	2014	Retrospective cohort analysis	25	Pediatric patients (mean age 13.5 years)	Posterior spinal fusion for spinal deformity	Topical application of vancomycin powder in pediatric spinal deformity surgery provides local antibiotic concentration above MIC for at least two days postoperatively without reaching toxic serum levels
Brown et al. [23]	USA	2004	Prospective randomized study	83	Adult patients	Extensive lumbar spine surgery	The decision to use or not use a wound drain following extensive lumbar spine surgery should be left to the surgeon's discretion as it does not significantly alter infection, hematoma, or neurological deficit rates

Infection Rates

The reviewed studies provided mixed results regarding the impact of spinal drains on postoperative infection rates (Table 3) [7,8,11-13,15,20]. In one- and two-level cervical spine fusions by Poorman et al. (2014) [7], no significant difference was observed in infection rates between patients with drains and without drains. Similarly, in the meta-analysis by Liu et al. (2016) [11], there was an insignificantly different rate of overall infections (P = 0.83) between patients in the drain and no-drain groups and a subgroup of only posterior spinal surgeries. On the contrary, in a study by Herrick et al. (2018) [8], a lower incidence of surgical site infection reoperation was found in the drain group when undergoing posterior cervical decompression with instrumentation.

**Table 3:** Comparison of drain usage and outcomes in posterior spine surgery: infection rates, hematoma rates, and recovery outcomes SSI: surgical site infection

Author	Drain type	Surgery type	Infection rate (%)	Hematoma rate (%)	Complications and outcomes
Poorman et al [7].	Closed suction	Cervical spine fusions	No significant difference	Not reported	Longer operative and hospital stay with drains
Herrick et al. [8]	Variable	Posterior cervical decompression	Lower reoperation for SSI	Not reported	Drains may reduce SSI reoperations
Liu et al. [11].	Jackson-Pratt	Lumbar decompression	No significant difference	Higher in non-drain group	Improved exudate management with drains
Walid et al. [12].	Closed suction	Lumbar fusion	No significant difference	No hematoma reported	Higher postoperative fever and anemia with drains
Kim et al. [13].	Subfascial drain	Lumbar surgery	No significant difference	Not reported	Positive drain tip cultures in SSI cases
Shi et al. [15].	Time-driven removal	Lumbar fusion	Lower in drain group	Not reported	Fewer hospital stays with earlier drain removal
Pivazyan et al. [20].	Closed suction	Posterior spinal surgery	No significant difference	Not reported	Prolonged antibiotic use with drains does not reduce SSI rates

Hematoma Formation

Another important outcome measured was the rate of postoperative hematomas. In general, most studies didn't find there to be any significant differences in their rates between samples, as is the case in Choi et al. (2016) [3] and Elfiky et al. (2022) [9]. However, the study by Liu et al. (2016) [11] observed a greater number of patients with saturated dressing in the group without drains, favoring drainage to some extent in the treatment of wound exudate. Regarding symptomatic hematomas requiring reoperation, most studies reported no significant difference between the groups with and without drains.

Recovery Outcomes

Each study also looked at recovery results, including duration of hospital stay and complications after surgery. Poorman et al.'s (2014) [7] study showed that patients in the drain group had a longer operative and hospital stay time despite no notable difference in overall complication rates. Likewise, Adogwa et al.'s (2018) [19] study showed that following surgery for spine decompression and fusion, a group sample where drains were used resulted in a longer hospital stay duration than the group sample, where drainage systems were not used in fusion surgeries.

On the contrary, Shi et al. (2021) [15] showed that time-driven wound drain removal is associated with less drain output, total blood loss, earlier ambulation, and a decrease in the length of hospital stay.

Complications

Several studies reported on the complications associated with the use of spinal drains. Walid et al. (2012) [12] demonstrated a borderline significant increase in postoperative fever and a higher incidence of posthemorrhagic anemia in the drained group. Kim et al. (2023) [13] found that although drain tip cultures for surgical site infection cases had a high positivity rate, the positive predictive value was low, so it was of little use in predicting infections.

Anemia and higher transfusion rates were also observed more frequently in the drain groups, as reported by Walid et al. (2012) [12], raising concerns about the routine use of drains in spinal surgery.

Drains were associated with an increased hospital length of stay in certain studies, but in specific situations, they also helped in early ambulation and decreased total blood loss [15]. Drains have been associated with decreased total blood loss in specific clinical contexts. This paradoxical outcome arises from effective management of blood and fluid removal, which minimizes the need for additional surgical interventions often required to address complications from excessive bleeding or hematoma formation [15]. In specific situations, drains increased postoperative fever and anemia, with limited predictive value on infections from drain tip cultures [12]. In general, when considering the use of spinal drains in postoperative spinal surgery, there is a consideration to be made from a state of benefit and ostensible harm. Although they could be helpful in the early recognition of infection and the management of wound exudate, they are also associated with protracted hospital stays and some complications. The decision to use spinal drains would have to be individually oriented and choice-based on the needs of the patient and within the surgical context, balancing expected benefits and harms. More high-quality studies are needed to set definite guidelines for the use of spinal drains in various spinal surgeries.

Discussion

The use of spinal drains in postoperative management of spinal surgeries is an issue of wide debate. A literature review aims to elucidate the effect of spinal drains on infection, hematoma formation, and the general recovery of patients. Evidence from the studies presented for review showed variable efficacy and some drawbacks of spinal drains.

The infection rate in this matter was affected differently by the presence of a spinal drain. For example, Poorman et al., (2014) [7] and Liu et al., (2016) [11] found no significant difference in the infection rate of patients with or without the drains, indicating that spinal drains do not necessarily contribute to the risk of postoperative infection. Herrick et al. (2018) [8] in their series found surgical site infection reoperations to occur at a reduced rate in the drain group, which might otherwise become another tentative benefit of drains in reducing further surgical interventions due to infections. This variability in findings could be attributed to differences in study design, patient populations, or surgical techniques. A critical concern is that hematoma formation following spine surgery may result in severe complications, such as neurological deficits. Most studies, such as those by Choi et al. in 2016 [3] and Elfiky et al. in 2022 [9], could not find significant differences between the drainage and non-drainage groups for the hematoma rate. However, Liu et al. (2016) [11] indicated that the rate of saturated dressings in the no-drainage group was lower, suggesting that drains might contribute to the management of excessive wound exudate and prevent hematoma formation. The effect of spinal drains on recovery outcomes has been investigated, including the length of hospital stay and postoperative complications. Some studies found that patients with drains had a longer hospital stay. For instance, Poorman et al. [7] found in 2014 that the drain group had longer operative times and lengths of hospital stay. In another study published in 2018, Adogwa et al. [19] reported that with spinal decompression and fusion, patients with drains also had longer lengths of stay. Shi et al. [15] demonstrated the opposite in 2021: time-driven wound drain removal leads to shorter length of stay and earlier ambulation without increasing the incidence of surgical site infections or symptomatic epidural hematomas. These findings indicate that timing and management of drain removal are the two most important factors affecting recovery. There were some complications associated with spinal drains. Walid et al. (2012) [12] noticed that patients with drains had a higher rate of postoperative fever and post-hemorrhagic anemia. Consequently, Kim et al. (2023) [13] documented that even in the cases of surgical site infection, while the positivity rate for drain tip cultures was high, the positive predictive value remained low, indicating that it was of minimal use for the prediction of infections. These are the various complications that are apparent and critical to keep in mind when operating.

The innovative technique to decrease postoperative blood loss in patients undergoing lumbar scoliosis surgery was discovered by Liang et al. (2020) [21]. The researchers divided 60 patients into three groups: tranexamic acid (TXA), Gelfoam, and control. These researchers reported that retrograde injections of TXA through a drain followed by clamping for one hour were associated with significantly decreased postoperative blood loss compared to the control group. The TXA group had less total drainage and shorter times of drainage retention and hospital stay. In addition, the TXA group had higher hemoglobin and hematocrit at the time of discharge. There were no significant differences in coagulation parameters among groups, and deep vein thrombosis or pulmonary embolism did not occur in any cases. This research finally deemed that the method is effective and safe in reducing blood loss after incision and length of hospitalization following lumbar scoliosis surgery, hence making this approach very easy and practical for clinicians. In 2014, Armaghani [22] reflected on the application of vancomycin powder in the prevention of postoperative infections in pediatric patients with spinal deformity surgery. This was a single-center retrospective cohort study involving 25 patients who received vancomycin powder in one-gram doses during wound closure after any given surgery. The patients' serum and surgical drain vancomycin levels were measured immediately postoperatively and over the first two postoperative days. These results indicated that the serum vancomycin levels were markedly below the toxicity threshold compared to the drain levels, which were markedly above the minimum inhibitory concentration for most common pathogens, arguing for effective antibiotic activity at the local level without systemic toxicity. There were no deep wound infections or significant antibiotic-related complications, with only one patient developing a superficial wound issue, which was managed successfully.

Cabrera et al. (2023) [10] investigated the global practices of spine surgeons regarding the use of wound drains in open lumbar fusion surgeries. The study found that 80.5% of the surveyed surgeons prefer using drains, predominantly subfascial drains. The study also revealed that surgeon demographics significantly influence drain utilization. Surgeons aged 35-64 years were more likely to use drains, and those who used coaptive films for wound closure also showed a higher propensity for drain usage. Criteria for drain removal varied: 52.8% of surgeons removed drains based on duration, commonly on postoperative day two, while 27.7% removed them based on output, typically less than 50 mL daily. Regional differences were notable, with surgeons from Asia Pacific, Europe, and Latin America more likely to follow time-based removal, whereas less experienced surgeons preferred output-based removal. The findings highlighted a disconnect between clinical practice and evidence-based recommendations. 

The decision to use spinal drains should be individualized, taking into account the specific clinical scenario and patient characteristics. Primarily, they focus on their role in removing excess blood and fluid to prevent complications such as hematoma and seroma formation. In addition, drains can assist in managing wound exudate and may provide early indications of infection through abnormal fluid characteristics. However, drains are also associated with longer hospital stays and certain complications. Surgeons should weigh the potential benefits against the risks and consider factors such as the type of surgery, patient comorbidities, and the timing of drain removal.

This review has several limitations. The heterogeneity of the included studies, variations in surgical techniques, and differences in postoperative care protocols could affect the generalizability of the findings. Additionally, the retrospective nature of many studies may introduce biases, and the sample sizes in some studies were relatively small. Further high-quality, randomized controlled trials are needed to establish definitive guidelines for the use of spinal drains in spinal surgeries.

Surgical drains should be employed very judiciously, based on the risk factors of individual patients, such as the complexity of the surgery and the comorbidities of the patient, to elucidate the best postoperative management in spine surgery. Future research needs to be done with large-scale randomized trials to appropriately understand the role that drains have to play in various kinds of spinal surgeries and to establish some standardized protocols regarding their application. Further, adding innovative wound dressings, occlusive and antimicrobial, among others, to postoperative care practices could even further lower infection rates and improve the outcomes of wound healing. It would be further useful to explore new dressing technologies, such as incisional negative pressure wound therapy, for any long-term effectiveness and cost-effectiveness, especially for complex spinal surgeries. The need exists for customizing postoperative care with regard to individual conditions like age, BMI, and general status. We recommend further research to be pursued into personalized approaches to postoperative management, using predictive analytics for clinical decision-making regarding the application of drains and dressings in spinal surgery.

## Conclusions

The use of spinal drains in postoperative spinal surgery yields variable outcomes, with no consistent evidence supporting a significant reduction in infection or hematoma rates across different types of spinal surgeries. While some studies showed that drains may reduce reoperation rates for surgical site infections, particularly in posterior cervical surgeries, others found no clear benefit in infection prevention. Moreover, drains were shown to assist in managing wound exudate, potentially decreasing saturated dressings and improving local control of wound fluid, but at the cost of prolonged hospital stays in many cases. Complications such as postoperative fever, anemia, and higher transfusion rates were also observed more frequently in drain groups, raising concerns about their routine use. These findings suggest that the decision to use spinal drains should be carefully weighed, considering the individual patient’s risk factors, surgical context, and expected postoperative outcomes. The potential benefits, such as improved wound management and reduced risk of surgical site infections in specific procedures, must be balanced against the risk of increased complications and longer hospital stays. Given the heterogeneity in outcomes, further high-quality, randomized controlled trials are essential to establish standardized guidelines for using spinal drains in different spinal surgeries.
